# RBR E3 ubiquitin ligases: new structures, new insights, new questions

**DOI:** 10.1042/BJ20140006

**Published:** 2014-02-28

**Authors:** Donald E. Spratt, Helen Walden, Gary S. Shaw

**Affiliations:** *Department of Biochemistry, Schulich School of Medicine and Dentistry, University of Western Ontario, London, ON, Canada, N6A 5C1; †MRC Protein Phosphorylation and Ubiquitylation Unit, College of Life Sciences, University of Dundee, Dow Street, DD1 5EH, U.K.

**Keywords:** catalysis, structure, ubiquitination, ubiquitin ligase, ANKIB1, ankyrin repeat- and IBR domain-containing 1, BRcat, benign-catalytic, CCCP, carbonyl cyanide *m*-chlorophenylhydrazone, Cdk5, cyclin-dependent kinase 5, cIAP2, cellular inhibitor of apoptosis 2, CK1, casein kinase 1, CPH, Cul7, Parc and HERC2 proteins, CRL, Cul-RING-ligase, Cul, cullin, Eps15, epidermal growth factor receptor pathway substrate 15, FANCL, Fanconi anaemia, complementation group L, HDAC, histone deacetylase, HECT, homologous with E6-associated protein C-terminus, HOIL-1, haem-oxidized IRP2 ubiquitin ligase 1, HOIP, HOIL-1-interacting protein, IBR, InBetweenRING, LUBAC, linear ubiquitin chain assembly complex, MDM2, murine double minute 2, MIRO, mitochondrial Rho GTPase, NEDD, neural-precursor-cell-expressed developmentally down-regulated, NEMO, NF-κB essential modulator, NF-κB, nuclear factor κB, NZF, Npl4 ZNF, Parc, parkin-like cytoplasmic p53-binding protein, PINK1, PTEN-induced putative kinase 1, PKC, protein kinase C, RanBP2, RAN-binding protein 2, RBR, RING-BetweenRING-RING/RING1-BRcat-Rcat, Rcat, required-for-catalysis, RNF, RING finger protein, RWD, RING finger and WD repeat-containing, SH3, Src homology 3, SHARPIN, SHANK-associated RH domain interactor, SILAC, stable isotope labelling by amino acids in cell culture, SUMO, small ubiquitin-related modifier, TOMM70A, translocase of outer mitochondrial membrane 70 homologue A, TRAF6, tumour-necrosis-factor-receptor-associated factor 6, TRIAD, two RING fingers and a DRIL (double RING finger linked), UBA, ubiquitin-associated, UBE2L, ubiquitin-conjugating enzyme E2L, UIM, ubiquitin-interacting motif, Ubl, ubiquitin-like, ZNF, zinc finger

## Abstract

The RBR (RING-BetweenRING-RING) or TRIAD [two RING fingers and a DRIL (double RING finger linked)] E3 ubiquitin ligases comprise a group of 12 complex multidomain enzymes. This unique family of E3 ligases includes parkin, whose dysfunction is linked to the pathogenesis of early-onset Parkinson's disease, and HOIP (HOIL-1-interacting protein) and HOIL-1 (haem-oxidized IRP2 ubiquitin ligase 1), members of the LUBAC (linear ubiquitin chain assembly complex). The RBR E3 ligases share common features with both the larger RING and HECT (homologous with E6-associated protein C-terminus) E3 ligase families, directly catalysing ubiquitin transfer from an intrinsic catalytic cysteine housed in the C-terminal domain, as well as recruiting thioester-bound E2 enzymes via a RING domain. Recent three-dimensional structures and biochemical findings of the RBRs have revealed novel protein domain folds not previously envisioned and some surprising modes of regulation that have raised many questions. This has required renaming two of the domains in the RBR E3 ligases to more accurately reflect their structures and functions: the C-terminal Rcat (required-for-catalysis) domain, essential for catalytic activity, and a central BRcat (benign-catalytic) domain that adopts the same fold as the Rcat, but lacks a catalytic cysteine residue and ubiquitination activity. The present review discusses how three-dimensional structures of RBR (RING1-BRcat-Rcat) E3 ligases have provided new insights into our understanding of the biochemical mechanisms of these important enzymes in ubiquitin biology.

## INTRODUCTION

The post-translational modification of proteins with the covalent attachment of the 76-residue protein ubiquitin is a critical event that ultimately determines the fate of many proteins in the cell. This process, known as ubiquitination, is involved in a multitude of processes including cell-cycle progression, transcriptional regulation, DNA repair, signal transduction and protein turnover by the proteasome [1]. Ubiquitination involves the sequential transfer of an ubiquitin molecule through an enzyme cascade consisting of an ubiquitin-activating enzyme (E1), an ubiquitin-conjugating enzyme (E2) and an ubiquitin ligase (E3), until an isopeptide bond is formed between the C-terminus of ubiquitin and the ε-amino group of a lysine residue on a substrate protein. The E2–E3 combination controls the specificity of the target protein selected for modification, the site of attachment to the substrate protein, the length of the ubiquitin chain and the type of lysine linkage (i.e. Lys^11^, Lys^48^ and Lys^63^) made between the attached ubiquitin molecules [2].

**Figure 1:**
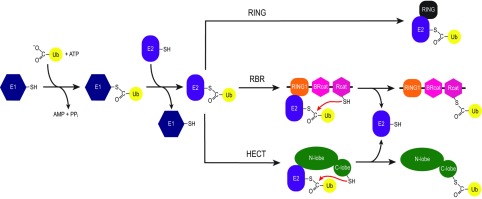
Proposed pathways for ubiquitination by different E3 ubiquitin ligases The ubiquitin activating enzyme (E1) activates ubiquitin through an ATP-dependent mechanism to form a thioester bond between the C-terminal carboxyl of ubiquitin and the catalytic cysteine in the E1. The ubiquitin is then transferred to an ubiquitin conjugating enzyme (E2) via a transthiolation reaction to form a thioester bond between the C-terminus of ubiquitin and the conserved catalytic cysteine residue of the E2. For the RING E3 ligases, the E2~ubiquitin complex engages with the RING domain of the E3 which optimally positions the ubiquitin in preparation for its transfer to a substrate protein. The HECT E3 ligases engage the E2~ubiquitin complex via their *N*-terminal lobe and perform another transthiolation reaction to form a thioester bond between the C-terminus of ubiquitin and the conserved catalytic cysteine residue in the C-terminal lobe of the HECT E3s. This HECT~ubiquitin intermediate is then poised for the subsequent transfer of ubiquitin to a substrate. The RBR E3 ligases use a combination of the RING and HECT mechanisms (termed a ‘RING–HECT’ hybrid mechanism [29]). In this mechanism, the RING1 engages with the E2~ubiquitin complex in a similar manner to the RING E3s, whereas the Rcat acts in a similar fashion to the C-terminal lobe of the HECT E3s by performing a transthiolation reaction to form a thiolester bond between the C-terminus of ubiquitin and the catalytic cysteine of the Rcat domain of RBR E3s.

There are different classes of E3 ubiquitin ligases that have been identified including RING, U-box and HECT (homologous with E6-associated protein C-terminus) E3 ligases (Figure 1). The RING and U-box E3 ligases function as scaffolds thought to orient the E2~ubiquitin thioester complex with respect to the target protein allowing for efficient ubiquitin transfer [2,3]. All RING E3 ligases co-ordinate two zinc ions via eight cysteine and histidine residues in a cross-brace formation [4], as exemplified from three-dimensional structures of c-Cbl [5], TRAF6 (tumour-necrosis-factor-receptor-associated factor 6) [6] and cIAP2 (cellular inhibitor of apoptosis 2) [7]. This fold positions conserved residues required for RING E3 ligases to engage with their cognate E2~ubiquitin and promote the transfer of the cargo ubiquitin to a target protein [8–11]. By contrast, HECT E3 ligases possess a common bilobal C-terminal HECT domain, and comprises an N-terminal lobe that retains the binding site for the E2 enzyme and a smaller C-terminal lobe that contains a conserved catalytic cysteine residue [12,13]. The HECT E3 ligases play a direct role in substrate ubiquitination by forming a catalytic intermediate thioester between the C-lobe cysteine residue and the C-terminus of ubiquitin [14–16]. Advances in our understanding of RING and HECT structures and mechanisms have been previously and excellently reviewed [2,3,17–19].

There is also an important group of E3 ligases known as the RBR (RING-BetweenRING-RING) or TRIAD [two RING fingers and a DRIL (double RING finger linked)] E3 ligases [20]. The best known of the RBR enzymes are parkin, which has a prominent role in the manifestation of early-onset Parkinson's disease, and HOIP (HOIL-1-interacting protein) and HOIL-1 (haem-oxidized IRP2 ubiquitin ligase 1), both of which are components of the multiprotein LUBAC (linear ubiquitin chain assembly complex). Unlike traditional RING- or HECT-style E3 ligases, all RBR E3 ligases identified to date are complex multidomain proteins. Initial sequence alignment methods suggested that two of the RBR domains contained multiple cysteine residues used to co-ordinate zinc ions that roughly conformed to the RING E3 ligase consensus sequence (RING1 and RING2) [20–22]. A third domain that lay between the proposed RING sequences, and again heavily populated by cysteine residues, was identified by multiple sequence alignment methods and aptly named an IBR (InBetweenRING) domain [23]. Thus the RBR nomenclature was born.

Initial experiments with several RBR E3 ligases including parkin and HHARI [also known as ARIH1 (Ariadne RBR E3 ubiquitin protein ligase 1)] were conducted on the basis that the RBR E3 ligases were in fact unusual E3 ligases that contained multiple RING domains and facilitated ubiquitination in a similar manner to the RING E3 enzymes [24–27]. However, recent advances in our understanding of the structural biology of RBR ligases, which are the focus of the present review, render the RING1-BetweenRING-RING2 nomenclature invalid. First, the **‘**RING2**’** domain of the RBR ligases does not conform to the canonical RING E3 structure; secondly, RBRs use an auto-inhibitory mechanism, first identified for parkin [28], that modulates ubiquitination activity; and thirdly, RBRs use a hybrid mechanism, first identified in HHARI [29], that combines aspects from both RING and HECT E3 ligase function to facilitate the ubiquitination reaction (Figure 1). Therefore we propose renaming the RBR domains while retaining the familiar RBR abbreviation as follows. The RING2 is not a RING, and possesses a single catalytic cysteine residue that allows it to accept an ubiquitin molecule from the E2 enzyme, form a thioester linkage with ubiquitin and transfer it to a substrate. As this domain is essential for RBR E3 ligase activity, a more appropriate naming should be a Rcat (required-for-catalysis) domain. The IBR domain, which we now know is actually not physically between two separate RING domains, adopts the same fold as the Rcat domain while lacking the catalytic cysteine residue and ubiquitination activity. Therefore this region can be more fittingly called a BRcat (benign-catalytic) domain. The present review will describe how new three-dimensional structures of RBR (RING1-BRcat-Rcat) E3 ligases have provided new insights into their ubiquitination biology and at the same time revealed many new unanswered questions.

## DOMAIN ARCHITECTURE OF THE RBR E3 UBIQUITIN LIGASES

**Figure 2:**
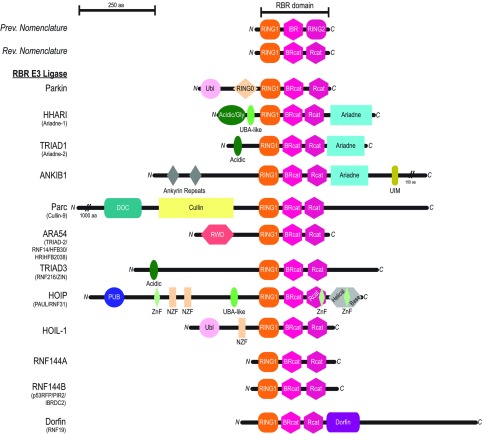
Domain architecture of the RBR E3 ubiquitin ligases Domains found in each RBR E3 ligase are RING1 (orange) BRcat (previously known as IBR; hot pink), and Rcat, (previously known as RING2; warm pink). Other domains listed include the Ubl (light pink), RING0 (wheat) and Npl4 NZF (wheat), acidic/Gly N-terminal extension (Acidic/Gly or Acidic; dark green), UBA-like (lime green), Ariadne domain (cyan), UIM (olive), ankyrin repeats (dark grey), docking domain (DOC; teal), Cullin (pale yellow), RWD (dark salmon), PUB (deep blue), ZnF (pale green), helical base (light grey) and Dorfin domain (deep purple). A conserved domain found in Cul7, Parc, and HERC2 proteins (CPH) is located in the N-terminal extension of Parc (not shown).

The overall domain architectures of the 12 RBR E3 ligases found in humans are illustrated in Figure 2. Intriguingly, to date no obvious examples of proteins have been identified that contain an isolated BRcat or Rcat suggesting that this triad of RING1, BRcat and Rcat domains are always found together in Nature. Furthermore, the RBR domains are invariably found in a particular order with the RING1 being sequentially followed by BRcat then Rcat [20,22], indicative that all three domains, including the BRcat, are required for RBR-mediated ubiquitination. However, the mechanism underlying ubiquitination is still unclear. In general, the RBR namesake of all human proteins is found near the C-termini of the E3 ligases, except for ANKIB1 (ankyrin repeat- and IBR domain-containing 1) and Dorfin where the RBR is located near the centre and N-terminus respectively. Interestingly, most RBR ligases contain a variety of different protein–protein interaction motifs near their N-termini. For example, both parkin and HOIL-1 contain N-terminal Ubl (ubiquitin-like) domains (Figure 2). The Ubl of parkin acts as an intramolecular auto-inhibitory domain by interacting with the RBR domain to attenuate ubiquitination [28] and has also been shown to bind to many other molecules including S5a [also known as PSMD4 (proteasome 26S subunit, non-ATPase, 4)] [30] and Eps15 (epidermal growth factor receptor pathway substrate 15) [30,31]. Likewise, the Ubl of HOIL-1 acts as a recruitment factor for HOIP through its N-terminal UBA (ubiquitin-associated) domain [32] to aid in the formation of the linear ubiquitin chain assembly complex, LUBAC. Parkin also has a unique cysteine-rich domain that was termed **‘**RING0**’** to fall in line with other domain nomenclature that is located immediately N-terminal to the RBR domain [33] and acts as a second inhibitory module by occluding the catalytic cysteine site in the Rcat domain [34–36]. Extended stretches of acidic residues are found near the N-termini of HHARI, TRIAD1 and TRIAD3 that were recently suggested to bind modified CRL [Cul (cullin)-RING-ligases] and cause RBR activation [37]. Other confirmed protein–protein interaction domain examples in RBRs include HOIP which has two NZF [Npl4 ZNF (zinc finger); NZF1 and NZF2) domains, where NZF1 binds to ubiquitin and NZF2 is required for SHARPIN (SHANK-associated RH domain interactor) Ubl recruitment [38], whereas HOIL-1 has one NZF domain that binds to linear ubiquitin chains with low micromolar affinity [38,39]. Numerous additional protein–protein interaction domains in the RBRs have been predicted including a PUB (PNGase/UBA- or UBX-containing domain; for binding to ATPase domain-containing proteins [40]), two ankyrin repeats in ANKIB1, an N-terminal RWD (RING finger and WD repeat-containing) domain in ARA54 [also known as RNF14 (RING finger protein 14)], as well as a conserved CPH [Cul7, Parc (parkin-like cytoplasmic p53-binding protein) and HERC2 proteins] domain involved in p53 binding [41] and a DOC (docking) domain in Parc.

In general, it appears that the C-termini of some of the RBRs are exclusively involved in auto-inhibitory interactions or controlling linkage specificity during ubiquitin chain assembly. For example, HHARI, TRIAD1 and ANKIB1 all contain Ariadne domains adjacent to their respective RBR domains that are involved in an intramolecular auto-inhibition mechanism whereby the Ariadne domain blocks access to the catalytic cysteine residue in the Rcat module [42], reminiscent of the mode of action used by the RING0 domain of parkin [34–36]. Interestingly, HOIP has a C-terminal extension of its RBR domain called a helical base that is responsible for the linear ubiquitin chain activity of the LUBAC [also known as the LDD (linear ubiquitin chain determining domain)] [43,44]. Furthermore, another unique feature of HOIP is the presence of two separate ZnF-like domains, with one in each of the Rcat and helical base domains, that are involved in forming a ubiquitin-binding platform required for linear ubiquitin chain building [43]. Finally, Dorfin has a unique namesake ‘Dorfin’ domain immediately C-terminal to its RBR domain [20]. This is suggestive that the Dorfin domain may be involved in modulating Dorfin's activity in a manner analogous to the inhibitory Ariadne domain in the Ariadne-containing RBRs [42]. Alternatively, it may possibly be involved in guiding ubiquitin chain linkage specificity like the helical base of HOIP [43–45].

## PROPOSED PROTEIN INTERACTIONS FOR RBR E3 UBIQUITIN LIGASES

Despite the identification of a large number of substrates, specifically in the case of parkin, we still know very little about RBR-mediated substrate recognition, how a substrate is ubiquitinated by an RBR E3 ligase and/or how the RBRs are regulated to control their ubiquitination mechanism. Furthermore, there are now several examples of previously identified RBR substrates and/or interacting proteins that cannot be reconciled with the recent structures of parkin and HHARI. A prime example is UbcH8 [UBE2L6 (ubiquitin-conjugating enzyme E2L 6)] that was originally shown to interact with the Rcat domain [26,46,47]. However, we now know that the Rcat is not a RING domain and that it lacks the conserved residues required for E2 recruitment [48]. Owing to its association with Parkinson's disease, parkin has been the most extensively studied of the RBRs and consequently the literature is biased towards proposed interacting proteins and substrates of parkin. In contrast, only a few interacting proteins/substrates have been observed for the other RBRs and the sites of interaction are not well defined.

Nevertheless, numerous proteins have been observed to interact with the RBR E3 ligases with some of these shown or predicted to be substrates for RBR-mediated ubiquitination. To help consolidate the literature and determine if there are any similarities between the RBRs and their interaction partners, we have assembled a comprehensive table of RBR interacting proteins that have been identified using direct experimental methods (Table 1). In general the predominant methods used to observe these interactions have been immunoprecipitation, yeast two-hybrid or pull-down experiments using N-terminal GST or His_6_ affinity tags. Many researchers have also used a variety of truncated proteins or protein fragments of the RBRs to further pinpoint the specific regions responsible for the observed interaction. Quantitative measurements have been sparse and are probably the next step in elucidating the molecular mechanisms employed by the RBRs to ubiquitinate their substrates.

**Table 1:** Observed protein–protein interactions with RBR E3 ubiquitin ligases Detection methods: 2H, yeast or mammalian-two hybrid; AUbA, autoubiquitination assay; CE, co-elution during chromatography purification; FRET, FRET *in vivo*; IF, immunofluorescence; IP, immunoprecipitation; ITC, isothermal calorimetry; LCMS, liquid chromatographyMS/MS; NMR, nuclear magnetic resonance; PD, pulldown using GST, His or MBP tag; Phos, *in vitro* phosphorylation; SPR, surface plasmon resonance; UbA, ubiquitination assay; UbSu, ubiquitin suicide inhibitor; X-ray, X-ray crystallography.

RBR E3 ligase	Interacting protein	Detection method(s)	RBR-interaction site	Reference(s)
Parkin	UbcH7 (UBE2L3)	IP, 2H, AUbA, UbA, SPR	RING1	[24,25,29,46,47,51,60,78,80,82,91–94,127,132,137–144]
	UbcH5c (UBE2D3)/Ubc7 (UBE2G1)/UbcH6 (UBE2E1)	IP, UbA		[93,140,144,145]
	UbcH8 (UBE2L6)/UbcH13 (UBE2N)	IP, PD, UbA	Rcat	[24,26,47,61]
	Ubiquitin-conjugating enzyme Variant 1a (Uev1a)	PD	RING1	[61]
	14-3-3η	IP, PD, UbA	RING0	[143]
	20S proteasome subunit α4 (PSMA7/XAPC7, subunit α type7)	2H, IP	BRcat–Rcat	[83]
	26S proteasome non-ATPase reg. subunit4 (Rpn10/S5a)	UbA, PD, NMR	Ubl	[30,79–82]
	α-Synuclein-interacting protein (Synphylin-1, Sph1)	IP, UbA, PD	Rcat	[94,141,143,145,146]
	All 1-fused gene from chromosome 6 (Afadin/AF-6)	IP, PD	Rcat	[147]
	Aminoacyl tRNA synthase complex coactivator (p38/JTV-1/AIMP2)	IP, PD	Ubl, RING1	[82,91,94,146,148–150]
	Apoptosis regulator Bcl-2	IP, PD		[151]
	Bcl-2-associated athanogene 5 (BAG5)	IP, PD, UbA		[152,153]
	Calcium/calmodulin dependent serine kinase (CASK/Lin2)	IP, PD, UbA	Rcat	[92]
	Carboxy terminus of Hsp70-interacting protein (CHIP)	IP, PD		[63]
	Casein kinase 1 (CK1)	IP, PD, Phos	Ser^101^, Ser^378^	[95,98]
	Catenin β-1 (β-catenin)	PD		[154]
	Chondroitin-polymerizing factor (ChPF/Klokin1)	IP, 2H		[155]
	Cul	IP, PD		[132]
	Cdk5	IP, PD	Ser^131^	[94,95]
	Cyclin E	IP, PD, UbA		[132,142,156]
	DJ-1 peptidase	IP, PD, UbA		[47,145,157,158]
	Dopamine transporter (DAT)	IP, PD		[64]
	E3 SUMO-protein ligase RanBP2	IP, 2H, UbA		[78]
	Endophillin-A1	PD, UbA, NMR	Ubl	[111]
	Eps15	PD, UbA, NMR, ITC	Ubl	[30,31]
	F-box/WD repeat-containing protein 7 (FBX30/SEL-10)	IP, PD	ΔUbl	[132]
	Heat-shock 70 kDa protein (Hsp70/chaperone protein DnaK)	IP	ΔUbl	[63,82,127]
	HDAC6	IP, PD	RING0, RING1, Rcat	[70,71]
	Leu-rich PPR motif-containing protein (LRPPRC, LRP130)	IP		[59]
	Leu-rich repeat kinase 2 (LRRK2)	IP	Rcat	[90]
	LIM kinase-1 (LIMK1)	IP, UbA	BRcat–Rcat	[91]
	Machado–Joseph disease protein 1 (Ataxin-3)	IP, UbA, PD, NMR	Ubl, BRcat–Rcat	[110,127,139,140]
	Mitochondrial Rho GTPase (Miro)	IP		[159,160]
	Mitofusin-1 & 2 (MFN1, MFN2)	IP, UbA		[49,51–57]
	Mortalin (HSPA9, GRP75, PBP74)	IP		[161]
	Neuronal DnaJ/Hsp40 chaperone HSJ1a (DNAJB2a)	IP		[162]
	O-glycosylated α-synuclein (αSp22)	IP, UbA		[60–62]
	Parkin-associated endothelin receptor (Pael-R)	IP, UbA		[46,63]
	Parkin co-regulated gene protein (PACRG/Glup)	IP		[163]
	Parkin-interacting substrate (PARIS/ZNF476)	IP, PD, UbA	RING1, Rcat	[144]
	Prolierating cell nuclear antigen (PCNA)	IP	RING1	[68,69]
	Protein interacting with C kinase 1 (PICK1/PRKCA BP)	IP, PD, UbA	Rcat	[93]
	Protein kinase A (PKA)	Phos	Ser^101^, Ser^131^, Ser^236^, Ser^378^	[98]
	Protein kinase C (PKC)	Phos	Ser^296^, Ser^378^	[98]
	PINK1	IP, UbA, Phos, PD, LCMS	Ser^65^, Thr^175^	[49,99–102,145,164–168]
	RNF41/NRDP1/FLRF	IP, 2H, PD, UbA	Ubl	[150,169]
	Septin4 (ARTS/CDCrel-2)/Septin5 (CDCrel-1/PNUTL1)	IP, 2H, PD	RBR	[26,82,83,138,170]
	Small ubiquitin-related modifer-1 (SUMO-1)	IP, PD		[77]
	Synaptotagmin XI (Syt11)	2H, IP, UbA	RING1	[171]
	TDP-43	IP, PD		[71]
	Transcription factor single-minded 2 (SIM2)	IP	BRcat–Rcat	[72]
	TOMM70A	IP, PD		[58,59]
	Tubulin (α, β & γ)	IP, PD, CE	RING0, RING1, Rcat	[82,172–174]
	Tyrosine protein kinase ABL1 (c-Abl)	IP, PD	Tyr^143^	[96,97]
HHARI (Ariadne 1)	UbcH7 (UBE2L3)	IP, 2H, IF, CE, ITC, PD, UbA	RING1	[27,29,37,42,114,175]
	UbcH8 (UBE2L6)	IP	Rcat	[27]
	α-Synuclein	IF		[176]
	α-Synuclein interacting protein (Synphilin-1, Sph1)	IF		[176]
	Cul-1,2,3,4A (NEDD8-dependent)	IP, UbSu	Acidic/Gly	[37]
	Transcription factor single-minded (SIM2)	IP		[72]
	Translation initiation factor 4F homologous protein (4EHP)	IP, 2H	RING1	[76]
TRIAD1 (Ariadne-2)	UbcH7 (UBE2L3)	IP, 2H, PD, PD, UbA	RING1	[37,177–179]
	UbcH6 (UBE2E1/UbcH8 (UBE2L6)/UbcH13 (UBE2N)	IP, 2H, PD	Rcat	[177–179]
	Cul-5 (NEDD8-dependent)	IP, UbA		[37]
	Growth factor independence 1 & 1B (Gfi1, Gfi1B)	IP, 2H, PD	Rcat	[180]
	MDM2	UbA		[84]
	Nuclear Inhibitor of NF-κB β (IκBβ)	IP		[181]
	p53	IP, PD		[182,183]
	Promyelocytic leukaemia-retinoic acid receptor α (PML-RARα)	IP, IF		[179]
Parc (CUL9)	UbcH7 (UBE2L3)	UbA		[131]
	Cul-7	IP		[88,184]
	NEDD8	IP, LCMS	Cullin at Lys^1881^	[88,89]
	p53	IP, PD, IF, CE, NMR	CPH	[41,88,131,185]
	Ring box protein-1 (Rbx1)	IP		[88]
ARA54 (RNF14, HRIHFB2038, HFB30 and TRIAD2)	UbcH6 (UBE2E1)/UbcH8 (UBE2L6)/UbcH9 (UBE2E3)	2H, AUbA	RING1	[186]
	Androgen receptor	IP, 2H, PD, SPR, FRET, X-ray	C-term FXXL(F/Y)	[73,74,187–194]
	Heterogeneous nuclear ribonucleoprotein A1 (hnRNP A1)	2H, M2H, PD		[195]
	p300/CBP-associated factor	2H		[188]
	T-cell factors 1 & 4 (TCF1, TCF4)	IP, PD		[196]
TRIAD3 (RNF216, ZIN)	UbcH7 (UBE2L3)/UbcH8 (UBE2L6)	IP		[197]
	Killer cell Ig-like receptor (KIR) 2DL4	IP, 2H	RBR	[198]
	Receptor interacting serine/threonine-protein kinase-1 (RIP1)	IP		[103]
	TNF receptor-associated factor 3 (TRAF3)	IP	N-term PXQX(T/S)	[199]
	Toll/interleukin-1 receptor adaptor protein (TIRAP)	IP		[103]
	Toll-like receptors 3,4,5, and 9 (TLR3,4,5,9)	2H		[197]
	Virion infectivity factor (Vif) of HIV-1	IP, PD		[200]
HOIP (PAUL, RNF31)	HOIL-1	IP, PD, CE, UbA, SPR, X-ray, NMR	UBA (via HOIL-1 Ubl)	[32,38,43–45,134,201–203]
	Sharpin (Sipl1)	IP, CE, UbA	NZF2	[38,43,45,133,134,202–205]
	UbcH7 (UBE2L3)	UbA	RING1–BRcat	[38,44,45,201,206]
	UbcH5A (UBE2D1)/UbcH5B (UBE2D2)/UbcH5C (UBE2D3)	UbA	RING1–BRcat	[39,43–45,65,85,105,134,201]
	E2-25K (UBE2K)	UbA		[201]
	NEMO	IP, PD, UbA		[39,65,67,133,134,205–207]
	B-cell surface antigen CD40 (CD40)	IP		[207]
	Muscle-Specific receptor tyrosine Kinase (MuSK)	2H		[104]
	Nucleotide-binding oligomerization domain protein 2 (NOD2)	IP		[208,209]
	OTU domain deubiquitinase with linear link specificity (Gumby)	IP		[87]
	Polyubiquitin chains (Lys^63^>linear>Lys^48^)	IP, PD, ITC	NZF1	[38,66,210]
	Tumour necrosis factor receptor 1 signalling complex (TNF-RSC)	IP		[66,85,211]
HOIL-1	cIAP1/2	IP		[66]
	Nucleotide-binding oligomerization domain protein 2 (NOD2)	IP		[208,209]
	Polyubiquitin chains (linear>Lys^63^)	PD, SPR, X-ray, ITC	Ubl, NZF	[38,39,66,210]
	Protein kinase C (PKC)	2H, UbA	Ubl	[105]
	Retinoic acid-inducible gene 1 protein (RIG-1)	PD	NZF	[85]
	Suppressor of cytokine signalling 6 (SOCS-6)	IP, 2H	Ubl	[212]
	Tumour necrosis factor α-induced protein 3 (A20)	IP		[211]
	Tumour receptor-associated factor 2 (TRAF2)	IP		[66]
RNF144A	UbcH7 (UBE2L3)	2H		[213]
RNF144B (p53RFP/IBRDC2/PIR2)	UbcH7 (UBE2L3)/UbcH8 (UBE2L6)	IP	RBR	[214]
	Bcl-2 associated protein X (BAX)	IP		[215]
	CDK-interacting protein 1 (p21^WAF1^)	IP		[216]
	Leukaemic nucleophosmin protein (NPMc)	IP		[217]
	p53, p63, p73	IP		[216,218–220]
Dorfin (RNF19)	UbcH7 (UBE2L3)/UbcH8 (UBE2L6)	IP	RBR	[221]
	α-Synuclein interacting protein (Synphillin-1, Sph1)	IP, UbA		[222]
	Calcium-sensing receptor	IP, 2H	C-terminal extension	[223]
	Cu/Zn SOD1 (ALS mutants; G37R/H46R/G85R/G93A)	IP, UbA	C-terminal extension	[224–228]
	Ubiquitinated-substrates (not defined)	IP	RBR and C-terminal extension	[221]
	Valosin-containing protein (p97/Cdc48 homologue)	IP, IF, LCMS		[223,226]
	Vimentin	IF		[221]

### RBR interactions with receptors and other membrane-associated proteins

Currently, the widely held view of parkin's role in the cell is to regulate mitochondrial clearance and mitophagy [49,50]. Consistent with this role, identified substrates for parkin include the transmembrane GTPase mitofusins 1 and 2 [49,51–57], TOMM70A (translocase of outer mitochondrial membrane 70 homologue A) [58,59] and O-glycosylated α-synuclein [60–62]. Parkin is also a candidate for dopaminergic signalling through interaction with the GPCR (G-protein-coupled receptor) Pael-R (parkin-associated endothelin receptor) [46,63] and the dopamine receptor [64] further underpinning its role in Parkinson's disease. The LUBAC, made up of a pair of heterodimeric RBR proteins HOIP and HOIL-1 along with SHARPIN, is involved in the innate immune and inflammatory responses [65]. These processes are controlled by the LUBAC interaction with the tumour necrosis factor receptor-signalling complex [66] to synthesize linear ubiquitin chains, which ultimately causes the recruitment of NEMO (NF-κB essential modulator) to activate the NF-κB (nuclear factor κB) signalling pathway [65,67].

### RBR involvement in DNA repair and RNA processes

There is increasing evidence that the RBR E3 ligases target DNA–protein complexes upon DNA breakage and DNA packing. For example, parkin interacts with PCNA (proliferating-cell nuclear antigen) [68,69] in damaged DNA as well as HDAC6 (histone deacetylase 6) [70] and TDP-43 (TAR DNA-binding protein 43) [71] involved in DNA packing. Parkin, HHARI and ARA54 also appear to be involved in transcription and translation. Interestingly, the transcription factor SIM2 (single-minded family bHLH transcription factor 2) can be ubiquitinated by parkin and HHARI [72]; however, the molecular basis for this processing by these RBR proteins is not known. Given the lack of conservation between parkin and HHARI outside of the RBR domains, is there a commonality between HHARI and parkin that enables two distinct RBRs to ubiquitinate the same substrate? Another example of an RNA-mediated process controlled by an RBR is ARA54 and its interaction with the transcription regulator androgen receptor, which is governed by the androgen receptor co-regulator signature FXXL(F/Y) motif found near the C-terminus of ARA54 [73,74]. However, the question of how ARA54 and ubiquitin directly regulate the androgen receptor is still unanswered. Likewise, the transcription factor 4EHP [also known as EIF4E2 (eukaryotic translation initiation factor 4E family member 2)], an mRNA cap-binding protein that contributes to the inhibition of 5′→3′ mRNA tethering [75], can be ubiquitinated by HHARI [76]. Perhaps the ubiquitination of 4EHP by HHARI causes an allosteric change or leads to the cellular turnover of 4EHP to allow for efficient protein translation? Future studies clarifying and expanding on the role of RBRs in DNA repair and RNA processes are needed.

### RBRs interacting with other ubiquitination machinery

There are numerous reports of RBR interactions with other ubiquitination pathway members. For example, parkin interacts with SUMO-1 (small ubiquitin-related modifier 1) and this association appears to modulate the activity of parkin as well as enhancing the import of parkin into the nucleus [77]. Likewise, parkin associates with and ubiquitinates the SUMO E3 ligase RanBP2 (RAN-binding protein 2) [78]. Parkin-mediated turnover of RanBP2 directly affects the intracellular levels of the SUMOylated histone deacetylase HDAC4 [78], an enzyme involved in DNA packing and transcriptional regulation. Taken together, these observed parkin interactions with SUMO-1 and RanBP2 further support a role for parkin in DNA and RNA processes. Parkin can also interact with 26S proteasomal subunits through its Ubl domain [30,79–82] and 20S proteasomal subunits through its BRcat and Rcat domains [83], suggesting different modes of interaction and/or recruitment can occur between the RBRs and the proteasome. TRIAD1 is another example, as it can interact with the E3 ligase MDM2 (murine double minute 2); however, in this instance, TRIAD1 is actually a substrate of MDM2 [84]. A suggested reason for TRIAD1 being ubiquitinated by MDM2 is to control p53 apoptosis signalling through balancing TRIAD1-dependent activation of p53 and MDM2-mediated destabilization of p53 [84]. HOIP has also been shown to interact with the ISG15 (interferon-induced 15 kDa protein) E3 ligase TRIM25 (tripartite motif-containing 25) [85], an enzyme implicated in the innate immune response against viral infection [86], and Gumby, a linear deubiquitinase involved in modulating the Wnt signalling pathway [87]. Clearly, the RBR E3 ligases are proposed to regulate, or be regulated, by other ubiquitination pathway proteins involved in a multitude of cellular processes.

Another interesting and recent development is the observation that HHARI and TRIAD1 can interact with CRLs in a NEDD8 (neural-precursor-cell-expressed developmentally down-regulated 8)-dependent manner [37]. The RBR E3 enzyme Parc (also known as Cul9) also contains a Cul7-like domain that can bind to the typical CRL partners including Rbx1 (RING-box 1, E3 ubiquitin protein ligase) and NEDD8 [88,89]. Parc appears to have originated from a gene fusion event between an RBR Ariadne gene and *Cul7* gene [20,22].

### RBR regulation by kinases

Parkin, TRIAD3, HOIP and HOIL-1 are the only RBRs to date that have been proposed as potential targets of protein kinases. For example, parkin can be phosphorylated by numerous kinases including LRRK2 (leucine-rich repeat kinase 2) [90], LIM kinase-1 [91], CASK (Ca^2+^/calmodulin-activated serine kinase) [92], PICK1 (protein interacting with PRKCA 1) [93], Cdk5 (cyclin-dependent kinase 5) [94,95], c-Abl (tyrosine protein kinase ABL1) [96,97], CK1 (casein kinase 1) [95,98], PKA (protein kinase A) [98], PKC (protein kinase C) [98], and PINK1 (PTEN-induced putative kinase 1) [99], with each having preferential phosphorylation sites in the Ubl, RING0, RING1 and BRcat domains. With the exception of PINK1, where phosphorylation of Ser^65^ in the Ubl causes an increase in parkin activity [100–102], the aforementioned kinases generally appear to attenuate the activity of parkin, possibly though protein aggregation as demonstrated with Cdk5 and CK1 [95]; however, the molecular basis of this activity loss is still unclear. In the case of TRIAD3 and the LUBAC, the kinases identified {RIP-1 (receptor-interacting serine/threonine-protein kinase-1) for TRIAD3 [103], MuSK (muscle, skeletal, receptor tyrosine kinase) for HOIP [104] and PKC for HOIL-1 [105]} have only been observed by immunoprecipitation and yeast-two hybrid experiments, and, to date, the sites of phosphorylation have not been identified and their downstream effects are still unknown. Does the phosphorylation of other RBRs also cause the loss of RBR ubiquitination activity due to aggregation? Future studies will hopefully further clarify the role of kinases in RBR regulation.

### In search of RBR substrates using MS

Recent MS studies have reported numerous parkin-binding proteins and substrates [59,106,107]. In one of these studies, MS in combination with SILAC (stable isotope labelling by amino acids in cell culture) and mitochondrial depolarization with CCCP (carbonyl cyanide *m*-chlorophenylhydrazone) was used to induce parkin recruitment to the mitochondria. As a result, ~90 different proteins with modified concentration levels were identified [106]. These included increased concentrations of proteins related to autophagy and the ubiquitin proteasome system, as well as decreased concentrations of outer mitochondrial membrane proteins of known parkin substrates involved in mitophagy including mitofusins 1 and 2, TOMM70A, and MIRO1 (mitochondrial Rho GTPase 1) and MIRO2 [106]. Remarkably, another study used SILAC in combination with quantitative diGly capture proteomics to identify parkin-dependent ubiquitination targets and astoundingly found ~4800 non-redundant ubiquitination sites in ~1700 proteins [107]. Surprisingly, this observation is orders of magnitude greater in terms of potential parkin substrates and ubiquitination sites than the previous 15 years of research combined. These researchers also found that parkin predominantly associated with the proteasome and mitochondrial proteins in response to CCCP-induced depolarization. Finally, another group identified 203 possible parkin-binding proteins using TAP (tandem affinity purification) interaction screens with MS and confirmed two of their hits [LRPPRC (leucine-rich pentatricopeptide repeat-containing) and TOMM70A] by immunoprecipitation [59]. Taken together, there are some commonalities that can be drawn from these studies that further support the role of parkin in mitochondrial mitophagy. For example, numerous proteins involved in mitochondrial clearance including mitofusin 1/2, MIRO1/2, mitochondrial fission 1 protein [106,107] and TOMM70A [59,106,107] are all identified as parkin interactors/substrates. What is perplexing is how similar methods can come up with such large differences in the number of possible substrates for parkin; however, these exciting results do provide a possible roadmap for further investigations into parkin and its interacting partners in the cell.

With the advent of high-throughput MS studies to identify protein–protein interactions and their interaction networks, it will be important to verify these parkin interactors and substrates by other methods as well to increase the confidence that the screens are reliable and reproducible under different conditions. This also raises an interesting question: would a similar strategy using MS be appropriate to identify interacting partners and/or substrates for the other RBR E3 ligases?

## NEW STRUCTURES OF RBR E3 LIGASES

**Figure 3:**
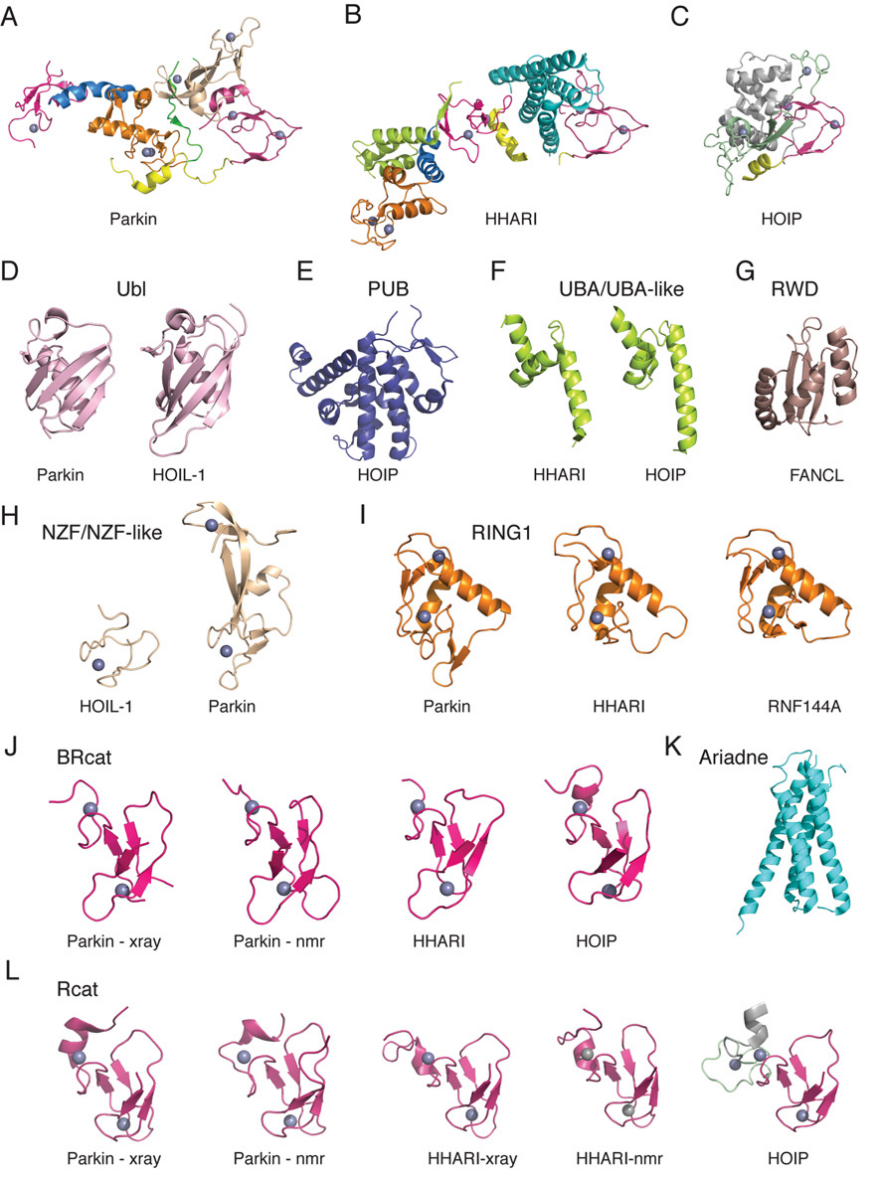
Catalogue of three-dimensional structures for RBR E3 ubiquitin ligases The upper panels show cartoon representations of multi-domain structures for (**A**) RING0–RBR from human parkin (PDB code 4I1F [35]; also PDB code 4K7D [34] and PDB code 4BM9 [36]), (**B**) human HHARI (PDB code 4KBL [42]) and (**C**) C-terminus of human HOIP (PDB code 4LJP [43]). The lower panels (**D**–**L**) show cartoon diagrams of three-dimensional structures of the individual domains for (**D**) Ubl domains from parkin (PDB code 2ZEQ [136]) and HOIL-1 (PDB code 2LGY [81]), (**E**) PUB domain from HOIP (PDB code 4JUY), (**F**) UBA-like domains from HHARI (PDB code 4KBL [42]) and HOIP (PDB code 4DBG [32]), (**G**) RWD from the E3 ligase FANCL (PDB code 3K1L [108]), (**H**) NZF and double NZF-like domains from HOIL-1 (PDB code 3B0A [39]) and parkin (PDB code 4I1F [35]), (**I**) RING1 domains from parkin (PDB code 4I1F [35]), HHARI (PDB code 4KBL [42]) and RNF144A (PDB code 1WIM), (**J**) BRcat domains from parkin (PDB code 4I1F [35] and PDB 2JMO [116]), HHARI (PDB code 4KBL [42]) and HOIP (PDB code 2CT7), (**K**) Ariadne domain from HHARI (PDB code 4KBL [42]), and (**L**) Rcat domains from parkin (PDB code 4I1F [35] and PDB code 2LWR [48]), HHARI (PDB code 4KBL [42] and PDB 2M9Y [117]) and HOIP (PDB code 4LJP [43]). The colour scheme for each individual domain and multidomain structures are as shown in Figure 2. Representative secondary structures are also labelled.

A wealth of three-dimensional structural information now exists for the RBR E3 ligases including multidomain and individual domain structures determined from X-ray crystallographic or NMR spectroscopic data. Multidomain structures include the RBR regions from parkin [34–36] and HHARI [42], and the C-terminal region from HOIP [43] (Figure 3). These structures have allowed for the juxtaposition of different regions to be assessed in terms of E3 ligase activity and have uncovered unique regions of each protein that alter catalysis (i.e. RING0 of parkin, Ariadne of HHARI and helical base of HOIP). In addition individual structures of many of the domains depicted in Figure 2 have been determined including the Ubl (parkin and HHARI), PUB (HOIP), UBA or UBA-like (HHARI and HOIP), NZF or NZF-like (HOIL-1 and parkin), RING1 (parkin, HHARI and RNF144A), BRcat (parkin, HHARI and HOIP), and Rcat (parkin, HHARI and HOIP) domains (Figure 3). Furthermore, the structure of the RWD domain present in ARA54 is expected to be similar in structure to that determined in other E3 ligases such as FANCL (Fanconi anaemia, complementation group L) [108] and RNF25 (PDB codes 2DAY and 2DMF). As described in the Introduction section, the structures of some RBR domains did not conform to expectations and, therefore, have provided new insights into their functions.

Although some of the domains are particular to an individual RBR protein, such as the PUB and RWD domains found in HOIP and ARA54 respectively, in general most of the domain structures are found in multiple RBR E3 ligases. Both parkin and HOIL-1 have an N-terminal Ubl domain and represent one of the earliest structures determined for the RBR ligases [79,109]. This domain shows the typical β-grasp fold for ubiquitin-type proteins and is expected to act as a protein-recruiting module. Multiple observations have shown the Ubl domain is able to interact with small motifs [UIMs (ubiquitin-interacting motifs), UBA domains and SH3 (Src homology 3) domains] with moderate affinity (10–400 μM). For example, parkin is able to interact with the UIM regions in the S5a proteasomal subunit [30,80], Eps15 [30,31] and ataxin-3 [110] as well as the SH3 domain of endophilin A1 [111]. Furthermore, the Ubl domain from parkin regulates E3 ligase activity in an auto-inhibitory fashion through interaction with its C-terminal RBR regions [28]. Structures and interaction studies show parkin utilizes the Ile^44^ face located on β3 to interact with all partners to date [30,110,111]. Interestingly, the HOIL-1 Ubl possesses an insertion between β1–β2 that is expected to lend specificity to this module [81]. This region and the C-terminus of helix α1 are used to form a surface on the opposite side from the Ile^44^ patch to recruit the UBA domain of HOIP, a requisite for linear polyubiquitin chain formation [32,81]. Although HOIP is also auto-inhibited for ubiquitination, these differences in Ubl structure and modes of interaction indicate its auto-inhibitory mechanism is not understood.

A common feature of the RBR E3 ligases is the presence of regions (UBA, NZF and ZnF domains) important for the recruitment of ubiquitin or polyubiquitin chains. Structures of the UBA domains from HHARI [42] and HOIP [32] appear very similar (RMSD=2.5 Å) yet neither appears to participate in interactions consistent with typical UBA domains (i.e. Dsk1 and PLIC [112]). For example, the HOIP UBA domain possesses a conserved **‘**GF sequence**’** between helices α1 and α2 yet uses an **‘**extra**’** α-helix to recruit the HOIL-1 Ubl domain [32]. Parkin, HOIP and HOIL-1 all have Zn^2+^-binding domains (NZF and ZnF) on the N-terminal side of the RBR module. HOIL-1 has been shown to have specificity for linear di-ubiquitin binding (*K*_d_ ~17 μM), whereby the distal ubiquitin interacts primarily with side chains from the NZF domain whereas the proximal ubiquitin utilizes an α-helix that lies C-terminal to the NZF domain [39]. The structure of the NZF from HOIL-1 reveals this domain co-ordinates a single Zn^2+^ ion via Cys_4_ co-ordination groups and has conserved tryptophan and asparagine residues that help maintain the protein fold as previously observed in the RanBP2 and Npl4 NZF domains [39]. The HOIL-1 NZF domain also follows the consensus sequence X_4_WXCX_2_CX_3_NX_6_CX_2_CX_5_ closely [113], as do the two NZF domains from HOIP, so these would be expected to have similar structures. On the basis of structural similarity with HOIL-1, it is not surprising that HOIP NZFs can also interact with ubiquitin [38], although the structural basis for this HOIP–ubiquitin interaction has not been shown yet. Originally missed in sequence comparisons, the discovery of the RING0 domain in parkin from limited proteolysis and MS experiments showed this protein also contained an additional Zn^2+^-binding domain [33]. It was recognized that the parkin RING0 domain would co-ordinate two Zn^2+^ ions in a linear fashion and that the N-terminal portion of RING0 retained some sequence similarity to the NZF domain in HOIL-1 [33]. Upon closer inspection (Figure 3H), it appears that that the second Zn^2+^-binding region in RING0 adopts a similar fold as the HOIL-1 NZF domain (RMSD=1.7 Å) using valine/glutamine residues in place of tryptophan/asparagine in the consensus and having a two-residue insertion within the second zinc-co-ordinating pair of cysteines. Furthermore, even though the first Zn^2+^ site in parkin is non-contiguous, the arrangement of the metal ion-co-ordinating residues also fits the NZF fold for HOIL-1 (RMSD=1.7 Å), although this site uses Cys_3_His co-ordination in parkin. On the basis of this structural comparison, it appears as though the RING0 domain has an unusual double NZF-like fold. With these insights it is perhaps not surprising that this double NZF-like structure has been shown to interact with ubiquitin using peptide array experiments [28]. However, the biological consequences of this interaction and those for HOIL-1 require further investigation.

**Figure 4:**
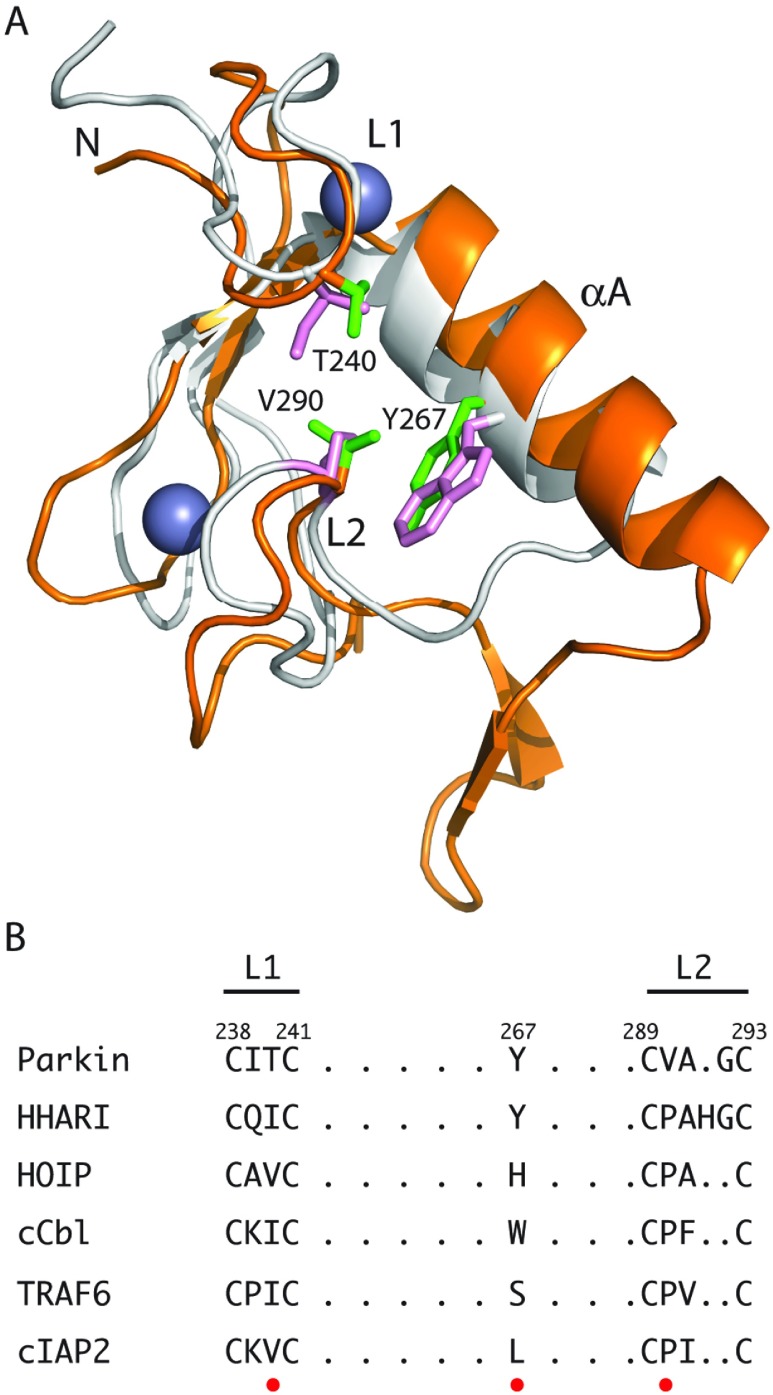
Comparison of RING domain structures for RBR and canonical RING E3 ubiquitin ligases (**A**) The structures of the RING1 domain from parkin (PDB code 4I1F [35]; orange) is superimposed with the RING domain from c-Cbl (PDB code 1FBV [5]; grey). The superposition was done using the C_α_ positions of the eight Zn^2+^-co-ordinating residues in each protein. The two regions (L1 and L2) in each protein and residues in parkin expected to be key for E2 interaction are indicated. (**B**) Sequence comparison for the RING1 domains of the RBR proteins parkin, HHARI and HOIP with representative RING E3 ligases c-Cbl, TRAF6 and cIAP2 showing important residues for E2 recruitment in L1 and L2 loops (red dot).

Based on the RING–HECT hybrid mechanism, the RING1 domain of RBR proteins is expected to be the E2-recruiting module [29]. Indeed several studies show that deletion or mutation of RING1 in parkin [26] and HHARI [114] leads to either decreased ubiquitination or interaction with the E2 enzyme UbcH7 (UBE2L3). These observations are consistent with structures of the RING1 domains from parkin [34–36], HHARI [42] and RNF144A which all show similar folds (RMSD=0.54–1.2 Å). Furthermore, the RING1 domains all adopt cross-brace Zn^2+^ ion co-ordination for two sites typical of other RING E3 ligase proteins such as TRAF6 [6] and c-Cbl [5] (Figure 4). There are some differences however between the RING1 domains in some of the RBR proteins that suggest E2 recruitment is perhaps not as straight-forward as in the canonical RING E3 ligases. For example, both parkin and HHARI have one or two extra residues within the L2 loop, a region shown to be important for interactions with E2 enzymes [17]. Furthermore, parkin contains a threonine residue rather than the traditional isoleucine/valine residue in L1 and lacks the highly conserved proline in L2 of the canonical RING E3 ligases [17]. It remains to be seen how these differences affect ubiquitination activity.

A low resolution structure (6.5 Å) of the C-terminus of parkin in complex with the Ubl domain has been modelled that shows that the Ubl domain interacts at a site near the L1/L2 region of RING1 [34]. Furthermore, structures of parkin also show the tether region (Figure 3A, shown in yellow) sits between these two loops and may interfere with E2 recruitment [34–36]. Perhaps as a result of this tether interaction with RING1, NMR studies showed poor affinity for parkin with UbcH7 that could be partly enhanced using mutations in the tether to disrupt its association with RING1 [34]. In contrast, direct binding experiments using surface plasmon resonance show much tighter binding of UbcH7 to both full-length parkin and parkin lacking the Ubl domain (*K*_d_ ~4–7 μM) [115]. It is interesting that despite very similar overall folds between the parkin and HHARI RING1 domains, HHARI shows affinity for UbcH7 between 200 and 500 nM using isothermal calorimetry [42], nearly an order of magnitude tighter than parkin.

The structure of the parkin BRcat domain shows a novel fold compared with other zinc-binding motifs where one Zn^2+^ ion is sandwiched between two pairs of β-strands and the second Zn^2+^ ion forms a gag-knuckle-type fold [116]. This linear zinc-binding arrangement is also found in HHARI [42] and HOIP (PDB code 2CT7). Although comparison of BRcat structures from multiple crystal and NMR structures provides a 0.8–2.0 Å RMSD between structures, the BRcat appears to be the most plastic of the three domains. In particular the β3–β4 loop appears to adopt multiple conformations in the NMR structure [116] and was poorly resolved in two of the three parkin crystal structures [35,36]. In HHARI and HOIP, this loop is 5–6 residues shorter, lacking several glycine residues found in parkin. Although first predicted to be a conduit between the RING1 and Rcat domains [116], the structures of parkin and HHARI show the BRcat occupies very different spatial locations in these two proteins - isolated in parkin and more central in HHARI (Figure 3). Thus although the conservation of the BRcat domain suggests it is more than a linker, the exact function of the BRcat domain remains a mystery.

**Figure 5:**
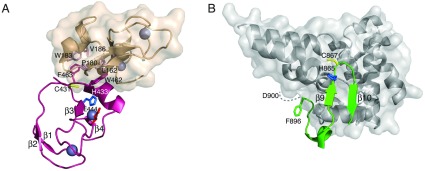
Similarity of catalytic sites for parkin and NEDD4 (**A**) The interface between the Rcat (pink ribbon) and RING0 (wheat surface) domains for parkin are shown highlighting important residues near the catalytic site. The three residues (Cys^431^, His^433^ and Glu^444^) important for ubiquitin transfer are shown in addition to several residues found at the Rcat (Trp^462^ and Phe^463^), RING0 (Lys^162^, Trp^183^, Pro^180^ and Val^186^) interface (PDB code 4I1F [35]). (**B**) A portion of the interface between the N-lobe (grey surface) and the catalytic region of the C-lobe (green ribbon) in NEDD4 is shown (PDB code 4BBN [122]). The catalytic cysteine (Cys^867^) resides between two β-strands similar to the position in parkin and HHARI. Two other residues important for catalysis (His^865^ and Asp^900^) are arranged in a mirror fashion compared to the Rcat domain in parkin and HHARI although Asp^900^ is not visible in the X-ray structure. In both structures the two β-strands were superimposed to achieve similar protein orientations.

Thought to form a canonical RING domain for many years, structures of the Rcat domain in parkin [34–36,48], HHARI [42,117] and HOIP [43] show that it adopts the same fold as the BRcat domain, an observation that was not originally predicted from sequence analyses. A significant difference is the presence of a conserved cysteine residue (Cys^431^ in parkin, Cys^359^ in HHARI and Cys^885^ in HOIP) in the Rcat domain that is required for ubiquitin transfer from the E2~ubiquitin conjugate to a substrate [29,35,44,45,48]. The Rcat domains show good agreement between multiple structures of parkin (RMSD=0.9–1.3 Å) and between different proteins (parkin–HHARI, RMSD=0.9 Å; parkin–HOIP, RMSD=1.6 Å). In the parkin, HHARI and HOIP structures, the catalytic cysteine is buried against the RING0 [34–36], Ariadne [42] and helical base [43] domains respectively, rendering the E3 ligases inactive. In all three cases hydrophobic residues at the extreme C-termini of the Rcat domains (Trp^462^ and Phe^463^ in parkin, Val^374^, Arg^391^ and Tyr^392^ in HHARI, and Met^886^ and Tyr^902^ in HOIP) mediate this interaction (Figure 5). It is interesting that NMR structures of the isolated Rcat domains from parkin [48] and HHARI [117] show some deviation in the position of this C-terminal region compared with the X-ray structures. Since it is expected that the Rcat interaction with the RING0 or Ariadne domains must be relieved in order to activate the E3 ligase, it is tempting to speculate that the position of the C-terminal helix and adjoining regions in the activated E3 ligase might take on the appearance of the position observed in the NMR structures of parkin and HHARI Rcat domains [48,117].

All structures show evidence of a catalytic triad comprising Cys^431^, His^433^ and Glu^444^ in parkin [34–36,48], Cys^359^, His^361^ and Glu^370^ in HHARI [42,117] and Cys^885^, His^887^ and Gln^895^ in HOIP [43]. *In vitro* experiments show that ubiquitin can be conjugated to the catalytic cysteine or the serine analogue and that substitution of any one of these residues renders the E3 ligase inactive [29,34–36,42,44,45,48], although cellular experiments show that Glu^444^ in parkin seems less important [35]. This would indicate that these residues are required for the loading and unloading of ubiquitin during a catalytic cycle. One proposal is that the histidine imidazole ring is polarized by the glutamate acidic side chain allowing the thiol group of the cysteine to become more nucleophilic towards the thioester linkage of the E2~ubiquitin donor [35,36]. For HHARI, this appears to have the largest effect on the unloading of the ubiquitin from the Rcat catalytic cysteine residue [42]. The structures partly support this idea whereby the imidazole ring is tipped towards the glutamate carboxylate and NMR data shows ND1 of His^433^ in parkin and His^359^ in HHARI are deprotonated [48,117], a requirement to hydrogen bond with the cysteine thiol side chain. Oddly however the cysteine side chain is pointed opposite to the histidine side chain (Figure 5) in a misaligned configuration similar to that observed in deubiquitinase enzymes [118–121]. Furthermore, in this configuration it is difficult to see how the cysteine side chain p*K*_a_ value would be altered to allow for thioester formation with the incoming ubiquitin protein. This indicates that binding of either the E2~ubiquitin, substrate or ubiquitinated substrate may have a role in the realignment and activation of the catalytic cysteine residue of the RBR E3 ligases.

The fact that the RBR E3 ligases are able to accept an ubiquitin from an E2~ubiquitin conjugate and form a short-lived Rcat~ubiquitin thioester prior to ubiquitin transfer to a substrate [29,35,44,45,48] parallels that for the HECT E3 ligases [12,13,15,16,122] (Figure 1). Intriguingly a comparison of the catalytic sites for parkin and HHARI show the arrangements of their catalytic residues are similar to that observed for a typical HECT E3 ligase. For example, the catalytic triads for both the RBR E3 ligase parkin and the HECT E3 ligase NEDD4 reside on anti-parallel β-strands and the intervening loop (Figure 5). Furthermore, the arrangement of the catalytic cysteine and histidine residues in parkin and HHARI appear to be a mirror image of that observed in NEDD4. The residue corresponding to Glu^444^ in parkin (Asp^900^ in NEDD4) is not observed in crystallographic data, but is required for catalysis [122]. Another interesting observation is the close presence of the N-lobe in NEDD4 to the catalytic site, a similarity to the RING0 domain in parkin (or Ariadne in HHARI).

## FLEXIBILITY AND CONFORMATIONAL CHANGES NEEDED FOR CATALYSIS

**Figure 6:**
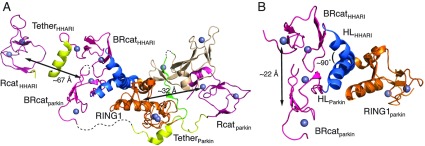
Evidence for flexibility in the RBR E3 ubiquitin ligases (**A**) The three-dimensional structures of parkin (PDB code 4I1F [35]) and HHARI (PDB code 4KBL [42]) are shown following superposition of their RING1 domains. This presentation shows the Rcat domains for the two proteins are found at opposite ends of the respective structures with respect to the location of the RING1 domain with large distances between the RING1 and Rcat domains (~32 Å in parkin and ~67 Å in HHARI) that must somehow be bridged for ubiquitin transfer. Regions not modelled in the parkin structure, probably due to flexibility, are indicated by broken lines. For clarity the UBA-like and Ariadne domains from HHARI are not shown. (**B**) The position of the BRcat domains in parkin and HHARI are shown after superposition of the RING1 domains. The position of the BRcat domain deviates ~22 Å between the parkin and HHARI structures due to an approximate 90° difference in the tilt of the RING1–BRcat interdomain helix. Only the RING1 domain from parkin is shown for clarity. The colour schemes used in (**A**) and (**B**) are as described previously in Figures 2 and 3.

Structures of parkin, HHARI and HOIP show that the cysteine residue (parkin Cys^431^, HHARI, Cys^357^ and HOIP Cys^885^) in the Rcat domain essential for ubiquitin transfer is buried against the RING0 [34–36], Ariadne [42] and helical base [43] domains respectively. Multiple experiments have shown that truncated forms of parkin lacking the Ubl, RING0, or RING0–RING1 domains [34], or HHARI lacking the Ariadne domain [42], support robust ubiquitination. Together these observations support the initial experiments by Walden and co-workers [28,123] that showed parkin, and now other RBR E3 ligases, exist in an auto-inhibited state that must undergo significant conformational change to relieve interactions of the Ubl and RING0/Ariadne/helical base domains to support ubiquitination. A need for conformational change is also exhibited by the large distances (32 Å in parkin) between the RING1 domain, where the E2 conjugate enzyme is predicted to bind, and Rcat domain where the cata-lytic cysteine resides, that must be traversed to transfer the ubiquitin cargo in all RBR E3 ligases (Figure 6).

As described the structures of the individual domains within the RBR regions appear remarkably similar (Figure 3). Yet upon further inspection, there are remarkable differences for the proximity and orientation of RING1, BRcat and Rcat domains between parkin and HHARI (Figure 6). This may reflect different mechanisms of activation used during the ubiquitination cycle. Alternatively, the structures may provide hints about the innate flexibility within the RBR domain structure and offer a snapshot of the ensuing conformational changes required for activation. For example, the majority of the parkin structures have poor electron density or have high thermal factors for connecting loop regions including Ser^218^–Glu^221^ (RING0-RING1 linker), Gly^355^–Lys^358^ (BRcat), Ala^379^–Gln^389^ and Ala^406^–Lys^412^ (BRcat–Rcat tether [34–36]). Furthermore, multiple parkin models from a single crystallographic data set show an approximate 13–16 Å translation of the BRcat domain between models [34]. Comparing parkin and HHARI structures also shows large differences in the positions of the BRcat and Rcat domain (Figure 6). In parkin, the BRcat and Rcat domains are separated by approximately 65 Å (centre–centre), whereas in HHARI these domains are nearly 30 Å closer to each other. Indeed, it is remarkable that these two RBR proteins show completely different relationships between the three domains despite the high similarities between individual like domains. A major difference here is that the 145-residue Ariadne domain of HHARI forms a four-helix bundle that intercalates between the BRcat and Rcat domains (Figure 3B). In parkin, the RING1, RING1–BRcat helical linker and a portion of RING0 lie between the BRcat and Rcat domains, thus pushing the BRcat and Rcat domains further apart than in HHARI. The tilt of the RING1–BRcat helical linker, found in both parkin and HHARI, could also account for these observed orientation differences. In both cases, this helix is bent near its centre, but is rotated ~90**°** in HHARI compared with the parkin structure, giving rise to a different spatial location of the BRcat domain (22 Å centre–centre) with respect to the RING1 domains in the two proteins (Figure 6). This observation and its effect on the position of the BRcat domain suggest the RING1–BRcat helical linker may be a key player in dictating the conformational changes required for activating the RBR E3 ligases.

## ACTIVATION OF RBR E3 LIGASES

It is clear from recent structural and biochemical work on parkin [28,34-36], HHARI [37,42], TRIAD1 [37], and HOIP and HOIL-1 [44,45], that the RBRs are auto-inhibited by subtly different mechanisms. Each RBR must presumably have several binding partners to achieve their ultimate function of ubiquitinating a lysine residue on a target substrate. They must have a productive interaction with an E2 enzyme, and they must also come into proximity with a substrate. In addition, there is evidence that parkin [28,124] and HOIP [43,44] both interact non-covalently with ubiquitin as part of their mechanism. Therefore it is possible to imagine several modes by which auto-inhibition may be achieved either through blocking an E2- or substrate-binding site and/or some auxiliary protein-binding site. Recent advances partially answer the question of how activation is achieved. In the case of parkin, there are at least three forms of auto-inhibition: the Ubl domain in its wild-type form interacts with the rest of parkin and blocks self-ubiquitination [28,48]; a helical region in the tether between the BRcat and Rcat domains contains a tryptophan residue that docks into the proposed E2-binding site on RING1 [34–36]; and the RING0 domain packs tightly against the catalytic cysteine residue of the Rcat in at least one conformation [34–36]. It is not yet understood how either the BRcat–Rcat tether or RING0 domain will be prised from their binding slots in parkin to allow for E2 binding and/or release the Rcat catalytic cysteine residue to form a thioester with ubiquitin. Indeed, removal of the key tryptophan residue in the BRcat–Rcat tether activates parkin for auto-ubiquitination, yet the RING0–Rcat interaction is presumably still intact [34–36]. This suggests that E2 binding to the RING1 domain may induce some conformational change that influences the RING0–Rcat interaction. Similarly, a BRcat–Rcat fragment that retains the tryptophan residue, but has no RING0 or RING1 domain, is also highly active [125,126]. However, the inhibition achieved by the Ubl domain is relieved by pathogenic mutations within that domain [28], and by phosphorylation of Ser^65^ by the mitophagy-specific kinase, PINK1 [100–102]. In addition, several parkin-binding partners are recruited through the Ubl domain, including endophilin A1 [111], Eps15 [30,31], proteasomal subunits [30,80,127] and ataxin-3 [110,127], suggesting that parkin activation may be achieved via a target or substrate-binding mechanism. There are also multiple reported post-translational parkin modifications outside of the Ubl domain, including S-nitrosylation [128,129] and NEDDylation [130], which have been reviewed recently [123].

Although parkin may employ an E2-blocking mechanism for regulation of activity, the same does not seem to be true for HHARI. In its full-length context, HHARI interacts with UbcH7 with a dissociation constant of 540 nM [42], which is a significantly higher affinity than typically displayed between E2s and E3s in the micromolar range [2,3]. Meanwhile, the C-terminal Ariadne domain, unique to the HHARI/TRIAD proteins [20], sits in between the BRcat and Rcat domains blocking access to the catalytic cysteine in the Rcat and lowers the activity in full-length HHARI [42]. Removal of the Ariadne domain is sufficient to release HHARI activity, and addition of the Ariadne domain *in trans* restores inhibition [42]. How this domain is released in a cellular environment is as yet unclear. However, a recent study found that both HHARI and TRIAD1, both of which contain an Ariadne domain, are activated by interaction with the NEDDylated forms of the CRLs [37]. This interaction may provide the means in cells to activate Ariadne RBR E3 ligases. Interestingly, there is also a cullin homology domain in the RBR ligase Parc [131], and evidence to suggest parkin forms a complex with CRLs [132]. Thus there is potential for an as-yet-unappreciated general role for cullins in RBR ligase activation.

Unlike the other RBRs, the LUBAC uses a different mechanism of auto-inhibition; however, the molecular basis of this auto-inhibition is presently unclear and probably more complex. LUBAC contains two RBR-containing proteins, HOIP and HOIL-1 [38,65,133,134]. As with the other RBR ligases, the RBR and helical base domain, which is unique to HOIP, are sufficient to recapitulate HOIP activity [43–45]. However, full-length HOIP is inactive [44,45] and, although removal of the N-terminal 700 amino acids releases HOIP activity [44], it is not yet clear what intramolecular arrangements are involved in the auto-inhibition of HOIP. Nevertheless, *in vivo* data show that HOIP activity is released through its interaction with the other components of LUBAC, namely SHARPIN and HOIL-1 [38,133,134]. Interestingly, a recent study suggests that parkin can team up with the LUBAC to enhance linear ubiquitination of NEMO that is dependent on both parkin's Ubl domain and its RBR ligase activity [135]. Clearly, further structural and biochemical details of the entire LUBAC are needed to better understand its modes of regulation.

Differences in modes of RBR ligase auto-inhibition will probably be reflected by differences in modes of RBR activation. Understanding how these RBRs are activated, on a molecular level, is a major challenge in our present understanding of their function(s) and activity.
